# Crossmodal Processing of Haptic Inputs in Sighted and Blind Individuals

**DOI:** 10.3389/fnsys.2016.00062

**Published:** 2016-08-02

**Authors:** Patrice Voss, Flamine Alary, Latifa Lazzouni, C. E. Chapman, Rachel Goldstein, Pierre Bourgoin, Franco Lepore

**Affiliations:** ^1^Department of Neurology and Neurosurgery, Cognitive Neuroscience Unit, Montreal Neurological Institute, McGill University, MontrealQC, Canada; ^2^Centre de Recherche en Neuropsychologie et Cognition, Département de Psychologie, Université de Montréal, MontréalQC, Canada; ^3^Groupe de Recherche sur le Système Nerveux Central, Département de Physiologie and École de Réadaptation, Faculté de Médecine, Université de Montréal, MontréalQC, Canada; ^4^Département de Radiologie, Faculté de Médicine, Université de Montréal, MontréalQC, Canada

**Keywords:** blind, tactile perception, crossmodal plasticity, fMRI BOLD, discrimination task

## Abstract

In a previous behavioral study, it was shown that early blind individuals were superior to sighted ones in discriminating two-dimensional (2D) tactile angle stimuli. The present study was designed to assess the neural substrate associated with a haptic 2D angle discrimination task in both sighted and blind individuals. Subjects performed tactile angle size discriminations in order to investigate whether the pattern of crossmodal occipital recruitment was lateralized as a function of the stimulated hand. Task-elicited activations were also compared across different difficulty levels to ascertain the potential modulatory role of task difficulty on crossmodal processing within occipital areas. We show that blind subjects had more widespread activation within the right lateral and superior occipital gyri when performing the haptic discrimination task. In contrast, the sighted activated the left cuneus and lingual gyrus more so than the blind when performing the task. Furthermore, activity within visual areas was shown to be predictive of tactile discrimination thresholds in the blind, but not in the sighted. Activity within parietal and occipital areas was modulated by task difficulty, where the easier angle comparison elicited more focal occipital activity along with bilateral posterior parietal activity, whereas the more difficult comparison produced more widespread occipital activity combined with reduced parietal activation. Finally, we show that crossmodal reorganization within the occipital cortex of blind individuals was primarily right lateralized, regardless of the stimulated hand, supporting previous evidence for a right-sided hemispheric specialization of the occipital cortex of blind individuals for the processing of tactile and haptic inputs.

## Introduction

Previous work has shown the blind to possess superior auditory abilities, particularly in the spatial hearing (Lessard et al., 1998; Voss et al., 2004) and in the pitch domains (Gougoux et al., 2004). These abilities are believed to be subserved by crossmodal processing and structural changes within occipital cortex (Gougoux et al., 2005; Collignon et al., 2011; Voss and Zatorre, 2012). While occipital cortex has also repeatedly been shown to be recruited for tactile processing in the blind (Burton et al., 2004; Stilla et al., 2008; Amedi et al., 2010; Sathian and Stilla, 2010), whether this leads to enhanced tactile processing is less clear. For instance, while Van Boven et al. (2000) and Goldreich and Kanics (2003) demonstrated that blind subjects are better than sighted ones in discriminating the orientation of a grating applied to the finger tip, both Grant et al. (2000) and Alary et al. (2009) did not find any group differences using the same stimuli. Similarly, Heller (1989) found no difference between blind and sighted subjects in a texture discrimination task, whereas Alary et al. (2009) showed the blind to be superior when performing this type of judgment.

To further investigate the issue of superior tactile processing in the blind, we recently explored both tactile and haptic perception using a two-dimensional (2D) angle size discrimination task that was developed by Voisin et al. (2002), and shown to be dependent not only on cutaneous, but also on proprioceptive feedback (Voisin et al., 2002). While early blind subjects outperformed sighted ones (Alary et al., 2008), it remained unclear whether this behavioral enhancement is subserved by crossmodal processing within occipital areas. Moreover, since crossmodal processing has also been observed in sighted subjects when processing tactile stimuli (Zangaladze et al., 1999; Sathian and Zangaladze, 2002; Sathian, 2005), it is equally unclear whether differential crossmodal activation patterns would emerge between both groups of individuals. Therefore, the primary goal of this study was to address these questions by exploring the pattern of brain activation elicited by an adapted version of the 2D angle discrimination task, designed to be compatible with the physical constraints of functional magnetic resonance imaging (fMRI). This enabled a novel proprioceptive element to the study, as most prior imaging studies of blind individuals focusing on the somatosensory system used passive tactile tasks. Furthermore, this approach is in line a growing body of literature establishing the importance of studying cognitive processes and their underlying neural activity in more action-oriented paradigms to better mimic how individuals interact with the environment (Engel et al., 2013).

Although it is established that occipital cortex plays a key role in tactile processing in the early blind (Burton et al., 2004, 2010; Stilla et al., 2008; Amedi et al., 2010; Sathian and Stilla, 2010), an additional goal of the present study was also to address several outstanding questions relating to specific factors that may modulate the pattern of crossmodal recruitment observed in the blind. For instance, it is currently unclear if the crossmodal recruitment of occipital areas for haptic/tactile inputs follows the same lateralization patterns observed in the somatosensory cortex, where stimulation in one hand is primarily processed by contralateral cortical areas. Similarly, it is currently not known if and how task difficulty influences the occipital crossmodal recruitment associated with tactile/haptic processing. For instance, it could be hypothesized that easy discrimination tasks recruit typical somatosensory and parietal brain areas normally associated with tactile processing, and that only difficult tasks recruit the additional available occipital areas for further processing.

Consequently, the objectives of the present study were primarily threefold. The first was to identify the neural substrates of haptic 2D angle discrimination, and to determine whether they differed between blind and sighted individuals. Second, we aimed to elucidate whether the crossmodal recruitment in the early blind would display any lateralization effects based on the stimulated hand. Lastly, we aimed to ascertain if regions showing crossmodal recruitment would modulate their activity as a function of task difficulty. To address these goals, fifteen early blind and fourteen sighted control subjects underwent a fMRI scan while they performed a categorized 2D angle size discrimination. Four different angles were used: the standard reference angle (90°), one that was very difficult to discriminate and well below the discrimination threshold, one that was moderately difficult and was just above the individual psychophysical threshold angle (*IPT* angle) measured for each subject as assessed in a previous psychophysical study (Alary et al., 2008), and finally an angle that was easily discriminable and well above the discrimination threshold of each subject. Subjects performed the entire set of discriminations using both hands in two separate functional runs.

## Materials and Methods

### Subjects

Fifteen early blind subjects participated in the study (ages 23–53 years, mean 37.47 years; 10 males). The average age of onset of blindness was 3.09 years (range 0–11). In all cases, blindness was attributable to peripheral damage and led to total blindness in all but four subjects who had residual light perception. All were fluent Braille readers (all were right-handed, though three used their left-handed for Braille reading). Fourteen healthy control (sighted) subjects were also studied (one left-handed; ages 22–50 years, mean 28.57 years; 5 males). All control sighted subjects had normal or corrected-to-normal visual acuity. Handedness of subjects was assessed by the Edinburgh inventory. While there is a discrepancy regarding the male/female ratio in each group, there are, to our knowledge, no documented differences between males and females in terms of cortical tactile processing or crossmodal plasticity. All subjects were free of any neurological deficits and had no MRI contraindications. The study was approved by the Ethics and Research Committees of the *Centre Hospitalier de l’Université de Montréal* (Notre-Dame Hospital), the *Centre de Recherche Interdisciplinaire en Réadaptation (CRIR)*, and *the Institut Nazareth et Louis Braille*. All subjects gave their informed and written consent prior to participation in the study.

### Neuroimaging Parameters

The BOLD (blood-oxygenation-level-dependant) measurements were performed on a Siemens Magnetom Avanto (Erlangen, Germany) 1.5 Tesla scanner (eight channel head coil) using a multi-slice gradient echo-planar imaging protocol. The functional T2^∗^ data were obtained using the following parameters: TE = 50 ms, FA = 90°, matrix = 64 × 64, TR = 3000 ms. A total of 684 functional volumes were acquired per subject, and each one consisted of 35 slices with a slice thickness of 4 mm (4 mm × 4 mm × 4 mm). An anatomical image was obtained with a high-resolution T1 scan (TR = 22 ms, TE = 9.2 ms, FOV = 256 mm, matrix = 256 × 256, voxel size = 1 mm × 1 mm × 1 mm).

### Stimuli

**FIGURE 1:**
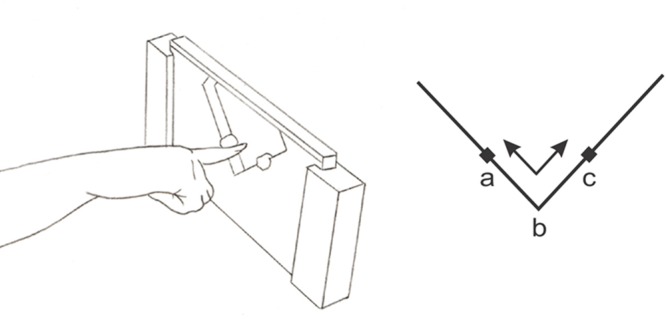
**Finger position during 2D-angle discrimination (90° reference angle shown here).** The angles were explored with the arm out-stretched using the distal phalanx of the index finger for exploration, the arm placed along the body, thus avoiding large shoulder or head movements. A single continuous to-and-fro movement was used by the subject to explore the angles during the stimulation block, following the sequence a-b-c-b-a (digit shown in the start position a here). The explored border was restricted to 3 cm on each surface, as delimited by plasticine markers placed just beyond the 3 cm point on either side of the intersection.

The stimuli were angles constructed from Plexiglas (**Figure 1**) and were formed by the intersection of two 8 cm long arms (for additional details, please consult Voisin et al., 2002). Two stops were placed 3 cm from the intersection, limiting the exploration to 3 cm over each arm of the angle, thus reducing elbow and shoulder movements. A range of angles was employed, including a standard reference angle of 90° (for the control condition), one that was below the discrimination threshold [91°; very difficult (*Different* angle) to discriminate from the reference angle], one that was suprathreshold [103°; easily (*Easy* angle) discriminable from the standard] and one that corresponded to the *IPT* angle for the subject (and thus varied from subject to subject). The IPT was determined based on the results obtained in a separate psychophysical testing session (Alary et al., 2008). The chosen IPT angle was the one closest, when rounded up, to the individual threshold previously determined. The IPT angles used here for the sighted subjects had a mean value of 6.75° ± 2.3°; those used for the blind subjects were smaller having a mean of 6° ± 3.1°.

### Stimulation Paradigm

Two separate functional runs were carried out with each subject: one where the angle exploration was performed by the right index finger and the other by the left (order counterbalanced across subjects). Within each run, all epochs followed a block design paradigm and were divided into blocks of 20 s of stimulation, followed by 12 s blocks of rest. The stimuli presented during a stimulation block was to one of the four possible angles corresponding to each condition: control (subjects explored the standard 90° angle), *Easy* angle (103° angle), IPT angle *Difficult* angle (using a 91° angle). The stimulation blocks were presented in a pseudorandom sequence (same sequence for all subjects) and each angle was presented eight times during the session, for a total of 32 presentations. Two separate sessions were performed: As such, a total of 64 stimulation blocks were presented to each subject, interleaved by 64 blocks or rest.

During image acquisition, the subject’s arm rested alongside their trunk. An fMRI compatible apparatus was placed in close proximity to the hand to be tested which held the angle upright. The subjects were instructed to keep their eyes closed for the duration of the experimentation. A screen placed outside the room such as to be seen only by the experimenter displayed instructions allowing the experimenter to change the angles following the sequences of stimulation and rest. For stimulation blocks, the experimenter placed the index finger of the subject at the start position (a, **Figure 1**) of the angle. The subject then explored the angle for 20 s, sliding the index finger back and forth (a-b-c-b-a) between the two stops as shown in **Figure 1**. This exploration involved mainly the wrist and fingers (corresponding to the distal exploratory strategy in Alary et al., 2008). For rest blocks, the experimenter withdrew the angle and the subject rested his/her hand next to the apparatus.

For each angle exploration, subjects were instructed to determine if the angle was equal to or superior to 90°, and to keep a running count of the number of angles judged to be >90°. This measure provided a convenient control to ensure that the subjects performed the task correctly, and was only communicated to the experimenter at the end of the session to minimize extraneous movements during data acquisition. Finally, before entering the fMRI room, the subjects tactually explored the 90° angle so that they could maintain a mental representation of it as the reference to which they would compare the angles presented during the fMRI acquisition. Subjects were not aware of which angles were to be used during the scanning protocol, and all subjects were instructed to keep their eyes closed during the entire time.

### Data Analysis

The imaging data were analyzed using Statistical Parametric Mapping (SPM8) software (Wellcome Trust Centre for Neuroimaging, London). Prior to statistical analysis, the functional images were realigned for each subject with the first image as reference to correct for head motion. Following the realignment, all images were normalized into an MRI stereotaxic space (MNI template of SPM8). Images were then convolved in space with a three-dimensional isotropic Gaussian kernel [8 mm full-width half-maximum (FWHM) for the individual analysis; 6 mm FWHM for the group analysis].

The analysis of functional data, based on a mixed-effects model, was then conducted in two serial steps, accounting, respectively, for fixed and random effects. For each subject, changes in brain regional responses were estimated by a general linear model including the responses to the four angle conditions (Control, Difficult, Easy, and Individual Psychophysical Threshold) and side of stimulation (left and right hand). The model consisted of a boxcar function convolved with the hemodynamic response function (hrf). High-pass filtering was implemented in the design matrix using a cutoff period of 128 s to remove slow drifts from the time series. Serial correlations in fMRI signal were estimated using an autoregressive (order 1) plus white-noise model and a restricted maximum likelihood algorithm. Linear contrasts tested the main effect of side stimulation (L, R), angle [Control (C), Difficult (D), Easy (E), and Threshold (Th) and group (B, S) statistical parametric maps were generated [SPM(T)]].

Statistical parametric maps of the bold response amplitude were then smoothed and subsequently entered in a second level analysis: a random effects model to test for inter-subject variance. We used a full factorial design where *F* tests characterized the main effect of group (B vs. S) and side of stimulation (L vs. R hand). Then, as the angle condition, displayed four levels we calculated T contrasts of angle differences (as pairs) to the control angle (D > C, E > C, and Th > C). A conjunction analysis based on a conjunction null hypothesis characterized brain areas activated jointly for the contrast D > C, E > C, and Th > C in both groups.

The resulting set of voxel values for each contrast constituted a map of the T statistic [SPM(T)], thresholded at *P* < 0.001 (uncorrected for multiple comparisons; Z threshold of 3.09) for the fixed effects analysis. Statistical inferences were performed at a threshold of *P* < 0.05 after correction for multiple comparisons over the entire brain volume for the random effects analysis. Significant clusters were anatomically labeled using structural neuroanatomy information using a brain atlas for brain regions provided by AAL toolbox under Matlab (Tzourio-Mazoyer et al., 2002).

## Results

### Behavioral Results

In a previous psychophysical study (Alary et al., 2008), we determined the individual 2D angle discrimination psychophysical threshold for each subject who participated in the present study. The mean threshold (75% correct) for the sighted subjects was 5.8° (SEM = 0.4, range 3.9°–8.7°), whereas the mean threshold was shown to be significantly lower for the blind subjects, at 4.9° (SEM = 0.8, range 1.6°–13.6°). For the current fMRI experiment, the IPT angle was selected from the available prefabricated series (see the section, Materials and Methods). After each fMRI session, the subjects verbally reported the number of angles counted as being >90°. The sighted subjects reported a mean of 14.5 angles that were >90° for both right and left finger explorations; the blind subjects reported a mean of 14.4 and 15.5 angles that were > 90°, for the right and left fingers, respectively. We had predicted that subjects would correctly identify all of the *Easy* angles (*n* = 8), 75% of the *Threshold* angles (6 of 8) and none of the *Difficult* angles (0 of 8), for a total of 14 (of the 24 comparison angles presented). The results are therefore in good agreement with this prediction, indicating that the subjects correctly performed the task. The observation that performance was slightly better than expected may reflect the fact that we opted to systematically use an angle just above the estimated threshold for the imaging sessions when an exact match was not available. This lead to an average deviation between the actual threshold and the angle used of 0.85 degrees for the blind (range: 0.1–1.47), and of 0.67 degrees for the sighted (range: 0.01–1.40). The difference between groups is not significant (*t* = 0.16, *p* = 0.87).

### Common Brain Activations Across and Differential Activations between Groups

**FIGURE 2:**
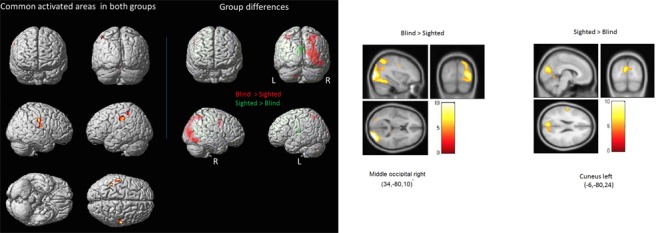
**Common activations across and differential activations between groups. (Left panel)** Highlighted on the left side are the areas that were commonly activated in both groups as indicated by a conjunction analysis, whereas those seen on the right side are areas that were significantly more activated in the blind (red) and those that were significantly more activated in the sighted (green) (see also **Table 2** for a complete list of areas). **(Right panel)** Illustrated here are the significant group differences again, shown in separate images to highlight the relative magnitude of the differences.

As illustrated in **Figure 2** and **Table 1**, several brain regions were commonly activated in both groups when performing the 2D angle discrimination task, as assessed by a group conjunction analysis. These regions are, not so surprisingly, those typically involved in tactile processing: the bilateral postcentral gyrus and the inferior parietal gyrus. When comparing both groups, the blind showed significant increases in brain activation in several areas (**Figure 2**), notably in the left inferior parietal gyrus, the right precentral gyrus, as well as the bilateral medial and lateral occipital gyri. In contrast, the sighted also showed significant BOLD increases in several cortical areas including the left cuneus and bilateral lingual gyri. In other words, both groups showed significant increases in activation within occipital areas that did not overlap as evidenced by group conjunction analysis. A complete list of differential activations can be found in **Table 1**.

**Table 1:** List of regions that showed significantly more activity in the blind compared to the sighted (top panel), showed significantly more activity in the sighted compared to the blind (middle panel), and regions that were equally activated in both groups as indicated by a conjunction analysis (bottom panel).

Brain region (label at cluster peak)	Cluster size (in voxels)	Hemisphere	MNI peak coordinates			*T*-values
			*X*	*Y*	*Z*	
**Blind > Sighted**				
**Middle/Lateral occipital gyrus**	**5205**	**Right**	**34**	**-88**	**10**	**11.76**
Precentral gyrus	471	Right	50	4	30	7.36
Inferior parietal gyrus	232	Left	-38	-60	54	6.83
Cerebellum	183	Left	-20	-66	-44	6.22
Superior frontal gyrus	39	Right	28	8	60	5.59
Medial frontal gyrus	44	Right	2	34	36	5.42
Thalamus	48	Right	8	-22	12	5.36
Thalamus	30	Left	-12	-20	18	5.34
Temporal inferior	66	Left	-54	-56	-16	5.29
**Middle/Lateral occipital gyrus**	**63**	**Left**	**-28**	**-88**	**10**	**5.24**
**Sighted > Blind**				
**Cuneus**	**1031**	**Left**	**-6**	**-80**	**24**	**7.82**
Postcentral gyrus	330	Left	-52	-18	16	7.33
**Lingual gyrus**	**289**	**Left**	**-14**	**-50**	**-6**	**6.95**
**Lingual gyrus**	**144**	**Right**	**10**	**-52**	**-2**	**6.48**
Superior temporal gyrus	27	Right	56	-32	14	5.73
Heschl gyrus	33	Right	54	-10	8	5.51
Parietal operculum	46	Left	-46	-2	14	5.42
**Middle/Lateral occipital gyrus**	**19**	**Left**	**-40**	**-60**	**4**	**4.99**
**Sighted ∩ Blind**				
Inferior parietal gyrus	127	Left	-56	-22	40	7.34
Postcentral gyrus	176	Right	54	-18	34	6.25
Inferior parietal gyrus	40	Left	-44	-40	54	5.53
Postcentral gyrus	14	Right	40	-28	38	5.31

*Bold values indicate brain areas located within occipital cortex.*

### Brain Activations Modulated by Stimulated Hand and by Task Difficulty

**FIGURE 3:**
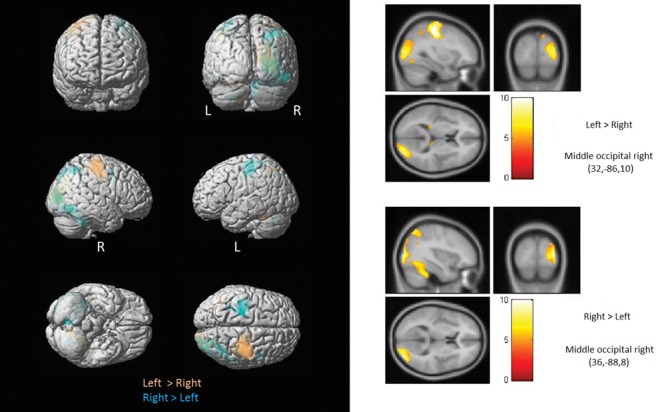
**Brain activations modulated by stimulated hand.** Shown here are regions showing increased activation in the blind as function of the stimulated hand. Compared to the sighted and regardless of the hand stimulated, the blind consistently showed increased activation of primarily the right occipital cortex (with only a small cluster showering increased activation in the left middle occipital gyrus in response to left hand stimulation).

**FIGURE 4:**
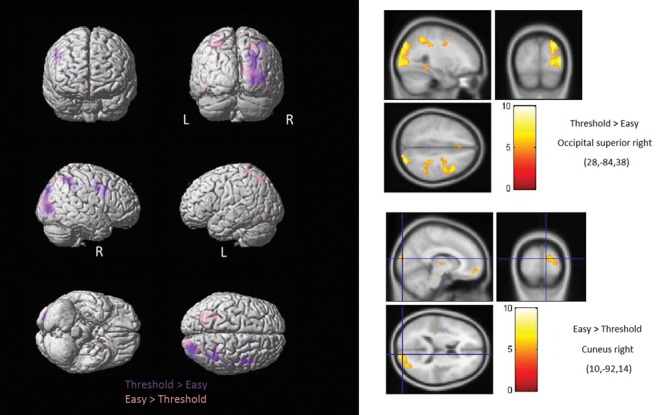
**Brain activations modulated by stimulated by task difficulty.** Shown here are regions showing increased activation in the blind as function of task difficulty. The Easy discrimination elicited more widespread bilateral activation in the parietal and occipital areas, whereas the Threshold (and more difficult) comparison yielded more activity primarily within the right occipital superior gyrus.

One of the primary objectives of the present study was to determine whether the crossmodal recruitment of occipital areas in the blind followed any specific lateralization patterns relative to the stimulated hand. Compared to the sighted and regardless of the hand stimulated (**Figure 3**; **Table 2A**), the blind consistently showed increased activation of primarily the right middle/lateral occipital gyrus (with only a small cluster showing increased activation in the left middle/lateral occipital gyrus in response to right hand stimulation). A second objective was to determine whether task difficulty modulated the crossmodal recruitment observed in occipital cortex. We therefore directly contrasted the blind > sighted contrasts obtained for the Easy and Threshold stimulus conditions. The easy discrimination eliciting more widespread bilateral BOLD activity primarily in the bilateral superior parietal gyri and the right superior occipital gyrus (**Figure 4**; **Table 2B**), whereas the Threshold (and more difficult) comparison led to more BOLD activity in the right angular gyrus and right superior occipital gyrus.

**Table 2A:** List of regions showing significantly increased activation in the blind as function of the stimulated hand.

Brain region (label at cluster peak)	Cluster size (in voxels)	Hemisphere	MNI peak coordinates			*T*-values
			*X*	*Y*	*Z*	
**Blind > Sighted (Right hand only)**				
**Middle/Lateral Occipital gyrus**	**2245**	**Right**	**30**	**-92**	**6**	**10.28**
Cerebellum	90	Left	-8	-76	-44	5.98
**Middle/Lateral Occipital gyrus**	**69**	**Left**	**-28**	**-82**	**8**	**5.74**
**Cuneus**	**13**	**Right**	**16**	**-86**	**14**	**5.62**
Angular gyrus	24	Right	36	-56	50	5.23
**Blind > Sighted (Left hand only)**				
**Middle/Lateral Occipital gyrus**	**1016**	**Right**	**18**	**-98**	**20**	**8.39**
Inferior temporal gyrus	131	Right	44	-66	-10	6.28
Precentral gyrus	91	Right	50	2	36	6.12
Inferior parietal gyrus	78	Left	-38	-58	54	5.88
Inferior parietal gyrus	132	Right	30	-54	46	5.71

*Bold values indicate brain areas located within occipital cortex.*

**Table 2B:** List of brain areas showing significantly increased activation in the blind as function of task difficulty.

Brain region (label at cluster peak)	Cluster size (in voxels)	Hemisphere	MNI peak coordinates			*T*-values
			*X*	*Y*	*Z*	
**Blind > Sighted (Easy > Threshold)**				
Superior parietal gyrus	928	Left	-32	-64	54	7.22
**Superior occipital gyrus**	**610**	**Right**	**14**	**-100**	**16**	**7.69**
Superior parietal gyrus	263	Right	28	-74	52	5.74
Inferior temporal gyrus	186	Right	48	-68	-10	6.28
Inferior temporal gyrus	178	Left	-48	-68	-8	7.12
Thalamus	132	Right	6	-18	6	5.86
Medial orbital frontal gyrus	107	Right	12	56	-14	6.78
Medial orbital frontal gyrus	94	Left	-12	14	-18	6.55
**Middle/Lateral occipital gyrus**	**83**	**Left**	**-30**	**-82**	**30**	**5.84**
Medial orbital frontal gyrus	13	Left	-10	66	-2	5.92
Postcentral gyrus	81	Right	28	-44	68	5.67
Precuneus	42	Right	6	-50	70	5.38
Precentral gyrus	32	Left	-24	-28	62	5.28
Medial orbital frontal gyrus	28	Left	-24	58	-10	5.8
Parietal operculum	25	Right	40	-28	18	5.41
Thalamus	10	Left	-18	-32	2	5.06
**Blind > Sighted (Threshold > Easy)**				
**Superior occipital gyrus**	**1600**	**Right**	**28**	**-84**	**38**	**8.59**
Angular gyrus	1125	Right	36	-50	50	8.45
Precentral gyrus	836	Right	52	10	30	7.47
Fusiform gyrus	175	Right	30	-42	12	5.39
Superior frontal gyrus	30	Right	26	14	58	5.56
Superior frontal gyrus	68	Left	-2	28	36	5.55
Superior frontal gyrus	67	Right	30	46	8	5.55
Postcentral gyrus	63	Right	56	-26	46	5.33
Cerebellum	34	Left	-30	-68	-28	5.03
Superior frontal gyrus	11	Right	18	-10	60	5.09
Postcentral gyrus	10	Right	22	-34	72	5.06

*Bold values indicate brain areas located within occipital cortex.*

**FIGURE 5:**
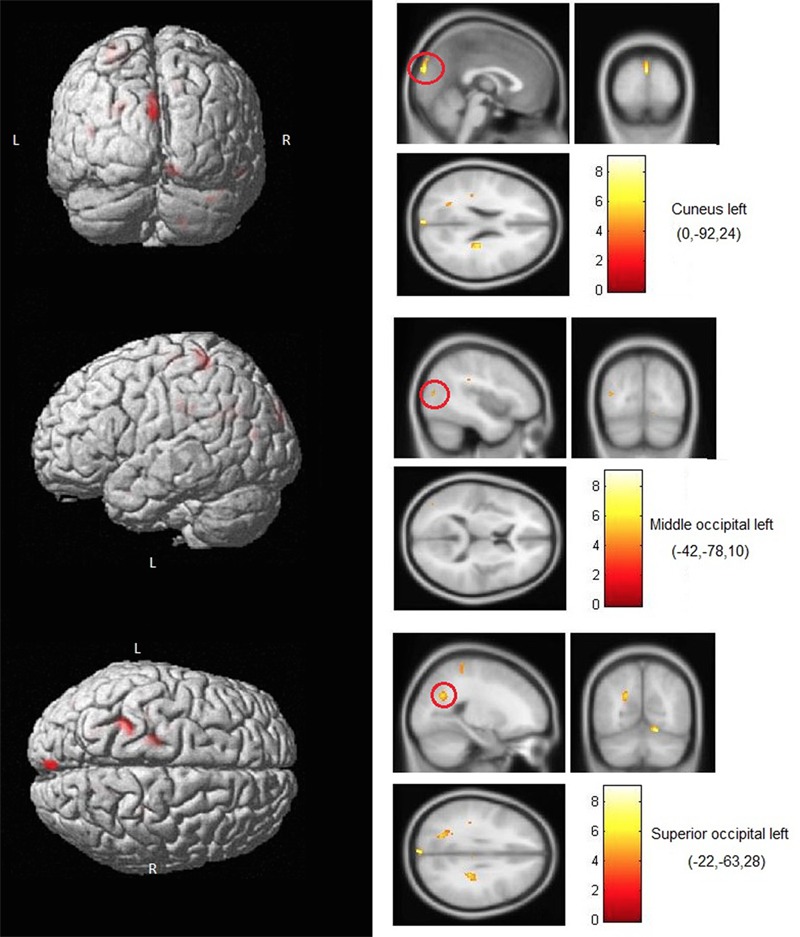
**Brain areas where activity predicted IPTs.** Shown here are occipital areas where BOLD activity during right hand exploration in blind subjects significantly correlated with the IPTs. No correlations involving occipital areas were found for left-hand stimulation or for either hand in the sighted subjects.

Finally, **Figure 5** (see also **Table 3**) illustrates the different occipital regions in the blind subjects for which the BOLD activation during right-hand angle exploration was shown to significantly predict the IPT of each subject. The resulting findings were obtained using voxel-wise regression analyses carried out across the entire brain volume using the IPTs as regressors. The same analysis was run for left-hand exploration in the blind, as well for both conditions in the sighted, and none of these analyses showed a link between occipital activity and IPTs.

**Table 3:** List of regions where activity during the IPT (threshold) condition was shown to significantly correlate with the individual thresholds in the blind group (for right hand stimulation only).

Brain region (label at cluster peak)	Cluster size (in voxels)	Hemisphere	MNI peak coordinates			*T*-values
			*X*	*Y*	*Z*	
Cerebellum	136	Right	16	-62	-14	9.1
Postcentral gyrus	110	Left	-24	-38	70	7.08
Insula	92	Right	30	-24	26	7.98
Hippocampus	87	Right	40	-24	-16	5.94
**Superior occipital gyrus**	**86**	**Left**	**-22**	**-64**	**28**	**5.77**
**Cuneus**	**76**	**Left**	**0**	**-92**	**24**	**7.8**
Inferior parietal gyrus	70	Left	-30	-26	34	5.76
Paracentral lobule	61	Left	-18	-26	66	5.12
**Lingual gyrus**	**34**	**Right**	**16**	**-34**	**-10**	**6.3**
Precuneus	22	Right	18	-52	42	4.8
Angular gyrus	11	Left	-26	6	-24	4.8
**Middle occipital gyrus**	**10**	**Left**	**-42**	**-78**	**12**	**4.8**

*Bold values indicate brain areas located within occipital cortex.*

## Discussion

The purpose of the present study was to examine the neural substrate underlying haptic discrimination of tactually explored 2D angles in sighted and visually deprived individuals. Two additional important goals were: (1) to determine if task difficulty had a modulatory effect on the observed crossmodal activity in occipital cortex of the blind, and (2) to determine whether the crossmodal recruitment of occipital areas followed the same contralateral lateralization principles as that observed for somatosensory areas. Regarding the first objective, we were able to show that in the blind, the haptic discrimination task elicited greater activation of occipital cortex compared to sighted subjects. Furthermore, we also found that the BOLD activity observed in several visual areas (e.g., cuneus and middle occipital gyrus) is predictive of the IPT for blind subjects; though this was only true for right-hand angle explorations. This unilateral effect is likely due to both the fact that all of the subjects were primarily right-handed and that the IPTs were measured with the right hand. It is reasonable to assume that we might have observed a similar correlational effect with left-hand exploration had we obtained IPTs for the left hand. It is of interest to note that the regions where BOLD best predicted IPTs do not completely overlap with the areas highlighted by the group contrast. This is likely because group contrasts are somewhat unspecific and hence reveal any differences that exist, whereas the use of thresholds as a regressor ensures task specificity, revealing only the regions showing a specific relationship with the computations elicited by the task. These findings are consistent with previous results in the auditory domain (Gougoux et al., 2005; Voss et al., 2008, 2014), and support the idea that this crossmodal recruitment likely underlies behavioral compensation in the blind.

We also showed that BOLD activity within parietal and occipital regions seemed to be modulated by task difficulty, where the easier comparison elicited more localized activity within the right lateral/middle occipital cortex and the bilateral posterior parietal gyri, whereas the more difficult comparison produced more widespread BOLD activity within the right superior and lateral/middle occipital gyri, combined with a reduction in parietal activation. Finally, we showed that crossmodal reorganization in occipital cortex of blind individuals was right lateralized, regardless of the stimulated hand. This suggests a hemispheric specialization of the right occipital cortex for the processing of tactile and haptic inputs that is independent of the lateralization of the input.

Overall, the crossmodal recruitment of occipital cortex in the blind in response to tactile discrimination was expected, and included the well-known lateral occipital complex (LOC) which is highly consistent with previous findings (Burton et al., 2004, 2010; Stilla et al., 2008; Amedi et al., 2010; Sathian and Stilla, 2010). Indeed, the LOC – which comprises part of the lateral occipital gyrus – has been repeatedly been shown to be involved in tactile exploration and processing in not only the blind, but also in sighted individuals (Burton et al., 2004, 2010; Stilla et al., 2008; Amedi et al., 2010; Sathian and Stilla, 2010). However, in the present study, there was very little overlap in the visual areas activated by both groups. While the blind recruited more heavily right occipital areas including the middle/lateral (LOC) and superior occipital gyri, the sighed in contrast showed increased activity in the left cuneus and lingual gyrus compared to the blind, closer to regions known for their role in orientation discrimination in the sighted (Zangaladze et al., 1999; Sathian and Zangaladze, 2002). The present data therefore argue in favor is a shift in the occipital regions involved in tactile and proprioceptive processing following prolonged visual loss. This hypothesis is further supported by the absence of occipital areas that were commonly activated in both groups (i.e., in the conjunction analysis).

The finding that right occipital areas were preferentially recruited compared to the left in the blind suggests a distinct hemispheric specialization of the right occipital cortex. While the finding of this right lateralization is not new (e.g., Burton et al., 2004, 2010), no previous study to our knowledge, had directly investigated the role played by the lateralization of the stimulated hand on the evoked crossmodal occipital responses. While Sadato et al. (1996) do show a similar right-sided activation via Braille reading with the right hand, they only note that the activation patterns were similar when elicited by left hand without actually presenting the data or without contrasting them. Similarly, Amedi et al. (2010) had their subjects explore objects tactually using both hands. However, they only compared the effect of handedness on activation observed within the LOC. Left occipital cortex appears to be preferentially recruited in the blind by tactile tasks containing important language components such as those typically evoked by Braille reading (Burton et al., 2002, 2012), which suggests that the right-lateralized responses seen here and elsewhere are likely driven by the spatial components of the tactile tasks.

The specific role played by the right middle and superior occipital gyri of the blind in the present task might be to perform general supramodal spatial computations. Indeed, these brain areas have also been shown to be heavily recruited by auditory spatial tasks in the blind (Gougoux et al., 2005; Renier et al., 2010; Collignon et al., 2011), and are also typically associated with visuospatial and motion processing abilities in sighted people (Haxby et al., 1991; Goodale and Milner, 1992). In fact, Renier et al. (2010) showed that the right middle occipital gyrus of blind individuals was preferentially recruited for spatial tasks in both the auditory and tactile domains compared to non-spatial tasks, without their being any difference in the activation patterns observed between sensory modalities. These findings along with the ones provided by the present study, strongly suggest that the right middle occipital gyrus of the blind likely plays a general-purpose supramodal role in the spatial processing of non-visual inputs.

We also investigated whether the crossmodal recruitment of occipital cortex in the blind varied as a function of task difficulty. This was done by comparing the group contrasts obtained by an Easy discrimination -where subjects were near 100% accuracy- with a slightly more difficult discrimination where subjects achieved nearly 75% accuracy (Threshold). Compared to the Threshold discrimination, the Easy discrimination recruited to a greater extent bilateral posterior parietal gyri in addition to the right superior and middle occipital gyri, whereas the opposite contrast revealed increased activation in only the right angular gyrus as well as both the superior and middle/lateral occipital gyri. These findings suggest two important implications: (1) the specific region within occipital cortex that is solicited for tactile discrimination varies as a function of task difficulty, and (2) there seems to be a trade-off or a shift in the computations being carried out between parietal and occipital cortex as the difficulty level increases. The latter implication is especially interesting, as it suggests that to solve easy tactile tasks the blind may only rely on parietal areas, whereas once the tasks become more difficult, they additionally solicit on occipital processing. This is further supported by significant correlations between BOLD in several occipital areas and the IPTs in the blind. This hypothesis is also highly consistent with previous findings in the auditory domain, where early blind individuals were shown to mostly activate parietal regions during an easy (binaural) localization task, and were shown to heavily recruit right occipital areas for a more difficult (monaural) sound localization task (Gougoux et al., 2005).

As highlighted earlier, the visual cortex also appears to play a role in tactile processing for sighted individuals *(*Zangaladze et al., 1999; Sathian and Zangaladze, 2002; Sathian, 2005). As such, the process by which occipital areas become increasingly responsive to tactile input after the loss of sight might be more straightforward than the often proposed ‘crossmodal takeover’ of visual areas for the processing of auditory inputs following blindness. Indeed, there is substantial psychophysical evidence that the somatosensory system computes stimulus features such as orientation of edges and motion in a similar manner as the visual system (Bensmaia et al., 2008; Pei et al., 2008, 2011). Furthermore, there is strong evidence that vision and haptics share spatial attentional resources in the sighted (Wahn and König, 2015) and that tactile and visual shape processing may rely on shared neural circuitry that extend beyond the occipital cortex (Yau et al., 2016).

## Conclusion

The present findings confirm previous results showing an involvement of deafferented visual areas in tactile processing, but also extend these findings in several ways. The task used here was an active exploration task (requiring both cutaneous and proprioceptive processing), which contrasts with the more passive tasks that have been often used before. This allowed us to study the neural substrate of tactile discrimination in a more action-oriented setting to better mimic how individuals interact with the environment (Engel et al., 2013).

## Author Contributions

PV, FA, CC, RG, PB, and FL designed the study. FA and RG collected data. PV, FA, LL, RG, CC, PB, and FL conducted data analysis. PV, FA, CC, LL, and FL wrote the manuscript.

## Conflict of Interest Statement

The authors declare that the research was conducted in the absence of any commercial or financial relationships that could be construed as a potential conflict of interest.
